# *N*^1^-methylnicotinamide promotes age-related cochlear damage via the overexpression of SIRT1

**DOI:** 10.3389/fncel.2025.1542164

**Published:** 2025-01-31

**Authors:** Toru Miwa, Akihito Tarui, Teppei Kouga, Yasunori Asai, Hideaki Ogita, Taro Fujikawa, Nobuhiro Hakuba

**Affiliations:** ^1^Department of Otolaryngology, Teikyo University Hospital, Mizonokuchi, Kawasaki, Japan; ^2^Department of Otolaryngology-Head and Neck Surgery, Graduate School of Medicine, Kyoto University, Kyoto, Japan; ^3^Department of Otolaryngology, Osaka Metropolitan University, Osaka, Japan; ^4^Department of Otolaryngology-Head and Neck Surgery, Fujita Health University, Toyoake, Japan

**Keywords:** age related hearing loss, Sirtuin 1, N^1^-methylnicotinamide, spiral ligament, metabolome, auditory brainstem responses

## Abstract

Age-related hearing loss (ARHL) is a complex condition with genetic, aging, and environmental influences. Sirtuins, particularly SIRT1, are NAD-dependent protein deacetylases critical to aging and stress responses. SIRT1 is modulated by nicotinamide N-methyltransferase (NNMT) and its product, N^1^-methylnicotinamide (MNAM), which influence ARHL progression. While SIRT1 is protective under certain conditions, its overexpression may paradoxically exacerbate hearing loss. This study examines MNAM supplementation’s impact on SIRT1 expression and ARHL in low-fat diet (LFD)-fed B6 and CBA mice. Mice were divided into LFD and LFD + MNAM groups and evaluated for auditory function, cochlear morphology, metabolic profiles, and SIRT1 expression at 3, 6, and 12 months of age. MNAM supplementation accelerated ARHL in both strains, with B6 mice showing more pronounced and earlier disease progression. Auditory brainstem response (ABR) thresholds were significantly elevated, and distortion-product otoacoustic emissions (DPOAE) indicated outer hair cell dysfunction. Cochlear histology revealed reduced hair cell and spiral ganglion cell counts, as well as decreased Na^+^/K^+^-ATPase α1 expression and endocochlear potential. MNAM increased SIRT1 protein levels in the cochlea without altering Sirt1 mRNA, suggesting post-transcriptional regulation. Metabolomic analysis revealed disrupted mitochondrial and oxidative pathways, including fatty acid oxidation and gluconeogenesis. Tricarboxylic acid (TCA) cycle dysregulation was evident, particularly in B6 mice, with elevated pyruvate, fumarate, and lactate levels. Despite similar metabolic trends in CBA mice, their slower aging profiles mitigated ARHL progression. These results suggest that while moderate SIRT1 expression protects against ARHL, overexpression disrupts metabolic homeostasis, accelerating cochlear aging and dysfunction. The dual role of SIRT1 emphasizes the need for precise modulation of its expression for effective therapeutic interventions. Future research should explore mechanisms underlying SIRT1-induced cochlear damage and strategies to maintain balanced SIRT1 expression. This study highlights MNAM’s detrimental effects on ARHL, underscoring its significance for developing targeted approaches to delay ARHL onset and preserve auditory function.

## 1 Introduction

Age-related hearing loss (ARHL) is a multifactorial condition resulting from genetic predisposition, aging, and cumulative disorders of the auditory system throughout an individual’s life (Lin, 2024). No preventative measures for ARHL have been established.

Sirtuins (Sirts) are a family of NAD + -dependent protein deacetylases that play critical roles in regulation of complex physiological processes, such as metabolism, aging, and cancer (Revollo and Li, 2013). Sirtuins also regulate apoptosis in response to oxidative and genotoxic stress in neurodegenerative diseases, including ARHL (Someya et al., 2009; Donmez and Outeiro, 2013). Sirt1 is particularly notable because its activity depends on NAD + availability, which links it to metabolic pathways that may influence age-related conditions (Chalkiadaki and Guarente, 2012).

### 1.1 Role of Nnmt and MNAM in Sirt1 regulation

Nicotinamide *N*-methyltransferase (Nnmt) influences energy metabolism by increasing NAD + levels and altering histone methylation (Kraus et al., 2014). Nnmt catalyzes the methylation of nicotinamide (Nam) to form *N*^1^-methylnicotinamide (MNAM), which demonstrates anti-inflammatory and anti-thrombotic effects and exerts a mitohormetic effect that extends the lifespan (Schmeisser et al., 2013). MNAM enhances SIRT1 protein expression, independent of its mRNA levels (Hong et al., 2015). Interestingly, calorie restriction (CR) upregulates Nnmt expression in C57BL/6 (B6) mice, thereby promoting SIRT3 expression and enhancing the mitochondrial antioxidant defense system, which in turn may delay the onset of ARHL (Someya et al., 2010).

Direct dietary supplementation with MNAM increases SIRT1 and SIRT3 protein expression in the cochlea (Miwa, 2021). Previously, we demonstrated that MNAM supplementation prevents high-fat diet (HFD)-induced and age-related hearing loss by enhancing SIRT1 and SIRT3 protein levels in the cochlea (Miwa, 2021). These findings suggest that SIRT1 and SIRT3 play essential roles in modulating ARHL progression.

### 1.2 Sirt1 in ARHL: contradictory evidence

Age-related increases in auditory thresholds and significant declines in SIRT1 expression have been observed in the cochlea and auditory cortex of B6 and CBA/J (CBA) mice (Keithley et al., 2004; Xiong et al., 2014; Takumida et al., 2016). In *Sirt1*-deficient B6 mice, auditory-evoked brainstem response (ABR) thresholds were significantly higher than those in wild-type controls, which highlights the potential role of Sirt1 in auditory function (IMPC).^1^ SIRT1 levels decline with age, and this decline contribute to ARHL (Tian et al., 2014; Xiong et al., 2015, 2019; Pang et al., 2019; Salam et al., 2021; Wang et al., 2023). In our previous study, we demonstrated that the expression of SIRT1 exhibited a greater age-related decline in mice subjected to a HFD compared to those on a LFD. Notably, the administration of MNAM significantly enhanced SIRT1 expression and conferred protective effects on cochlear structures (Miwa, 2021).

However, contradictory findings have complicated this narrative. Han et al. (2016) reported that partial SIRT1 loss in heterozygous *Sirt1* transgenic B6 mice reduced oxidative damage to hair cells and delayed ARHL onset. These results suggest that SIRT1 expression may influence ARHL in a dose-dependent manner, leaving it unresolved whether SIRT1 protects against or contributes to ARHL progression (Zhao and Tian, 2022; Koo et al., 2024).

Although these conflicting reports may be attributed to variations in experimental design, dosage, or animal models, the similarity of their methodologies to ours (Han et al., 2016; Miwa, 2021) underscores the need for further investigation into the effects of MNAM administration on SIRT1 expression.

### 1.3 Insights beyond the inner ear

Evidence from other systems adds complexity to the SIRT1-ARHL relationship. For example, Alcendor et al. (2007) reported that SIRT1 overexpression increased apoptosis in cardiac tissue, thereby reducing cardiac function; however, moderate SIRT1 expression in cardiac tissue protected against oxidative stress and enhanced catalase expression. Similarly, Li et al. (2008) demonstrated that SIRT1 inhibition protected cortical neurons from oxidative damage. These findings underscore the potential significance of moderate SIRT1 expression in balancing protective and deleterious effects.

### 1.4 Hypothesis and future directions

To date, no study has investigated the effect of moderate SIRT1 expression on ARHL. Based on previous findings, we propose that minimal, yet sufficient, SIRT1 expression may act as an anti-aging factor in cochlear cells. Achieving this optimal expression level could prevent ARHL progression, while avoiding the deleterious effects associated with SIRT1 overexpression. Future research should focus on elucidating the precise role of SIRT1 in the cochlea and determining whether moderate expression levels can effectively delay the onset of ARHL.

## 2 Materials and methods

### 2.1 Animals

Thirty 4-week-old male B6 and CBA mice (*Mus musculus)* were obtained from Shimizu Laboratory Supplies Co., Ltd. (Kyoto, Japan). Mice were randomly divided into two groups: low-fat diets (LFD) + MNAM (15 mice) and LFD (15 mice). Each group was further divided into subgroups based on age (3, 6, and 12 months; five mice per group). The body weights of the mice in each group were matched at the onset of the study. Animals were housed in air-conditioned rooms (25°C, approximately 50% humidity) with free access to feed and water. After a 1-week acclimation period, the mice received irradiated LFD (Low-Fat Diet 32, CLEA Japan Inc., Tokyo, Japan) or LFD supplemented with 1% MNAM (TCI America, Portland, OR, USA) (prepared by mixing MNAM powder at 1 wt./wt.) in accordance with previous studies (Hong et al., 2015; Miwa, 2021). Since water was provided *ad libitum*, its consumption was not measured. Food was administered weekly, and the remaining and discarded portions were recorded at each measurement to accurately determine food intake. All procedures were approved by the Animal Use and Management Committee (Kitano Hospital, Osaka, Japan; approval number A1900006), and the study adhered to the established guidelines.

### 2.2 Body weight and blood analysis

Body weights were recorded at 3, 6, and 12 months of age. Tail venous blood samples collected at 6 months were analyzed using a biochemical auto-analyzer™ (BioMajesty JCA-BM6050, JEOL Ltd., Tokyo, Japan).

### 2.3 Tissue preparation

Mice were euthanized via cervical dislocation, followed by cardiac perfusion with 4% paraformaldehyde (PFA). Inner ears were dissected, decalcified in 0.5M ethylenediaminetetraacetic acid/phosphate-buffered saline (EDTA/PBS), embedded in optimal cutting temperature (OCT) compound medium (Sakura Finetek Japan, Tokyo, Japan), and sectioned into 12 μm slices for analysis.

### 2.4 Immunohistochemistry

Primary antibodies include anti-SIRT1 (1:100; Proteintech, Rosemont, IL, USA) and anti-Na^+^/K^+^ ATPase α1 (1:200; Novus, Centennial, CO, USA). Samples were blocked using 10% goat or donkey serum, incubated with primary antibodies overnight at 4°C, and stained using fluorophore-conjugated secondary antibodies (1:500). Nuclear staining was performed using the Hoechst 33258 (Molecular Probes, Eugene, OR, USA). Fluorescence imaging was performed using a BZ-9000 microscope (Keyence, Osaka, Japan).

### 2.5 Hair cell counts

The surface morphology of the cochlea was examined after staining with Texas Red X phalloidin (1:100; Molecular Probes). Outer hair cells (OHCs) and inner hair cells (IHCs) were counted in 140 μm basal membrane segments at 40 × magnification across five randomly selected organ of Corti regions per cochlear turn. The counts for the whole length of the cochlea were estimated by extrapolating the data obtained from the 140 μm segments (Miwa et al., 2020). A second researcher verified the counts to ensure accuracy.

### 2.6 Spiral ganglion cell counts

Spiral ganglion cells (SGCs) were identified using anti-beta III tubulin (Tuj1) (1:200; Covance, Princeton, NJ, USA) and Hoechst staining. Cells positive for both markers in the Rosenthal canal were counted in three sections from the basal, middle, and apical cochlear turns (Yamada et al., 2015). ImageJ software (NIH, Bethesda, MA, USA) was used to prevent double counting.

### 2.7 Modified labeling index (mLI)

The modified labeling index (mLI) was used to quantify immunostaining in cryosectioned cochlear samples (Takeda et al., 2016, 2019; Miwa et al., 2021). Images were captured using consistent settings and processed using Adobe Photoshop (Adobe Inc., San Jose, CA, USA). The background intensity was measured using the Magic Wand tool, and stained regions were selected for optical density analysis. The final staining intensity was calculated by subtracting the background intensity, and the percentage of stained pixels was used to compute the staining ratios. All measurements were blinded, and five samples per group were analyzed.

### 2.8 Enzyme-linked immunosorbent assay (ELISA)

Mice were euthanized via cervical dislocation. Inner ears including lateral walls were dissected and preserved at −80°C. Cochlear protein lysates were prepared by homogenization of the samples using a Sonifier S-250A analog ultrasonic processor (Branson, Danbury, CT, USA) and quantified using a Mouse ELISA SIRT1 kit (Abcam, Cambridge, UK) in accordance with the manufacturer’s protocol. Absorbance was measured at 450 nm using a Multiskan FC microplate reader (Thermo Fisher Scientific, Waltham, MA, USA).

### 2.9 Quantitative real-time reverse transcriptase–polymerase chain reaction (qRT-pCR)

Mice were euthanized via cervical dislocation. Inner ears including lateral walls were dissected, permeated in RNA later (Thermo Fisher Scientific), and stored at −80°C. Total RNA was extracted using a QIAGEN kit (QIAGEN, Valencia, CA, USA) and Complementary DNA (cDNA) was synthesized using a One-Step PrimeScript RT-PCR Kit (Takara Bio, Otsu, Japan). *Sirt1* and glyceraldehyde-3-phosphate dehydrogenase (*GAPDH*) mRNA levels were quantified using the Takara Dice TP960 system. Expression levels were normalized to *GAPDH*. *Sirt1 mRNA* primer is Fw- TATCTATGCTCGCCTTGCGG and Rv- CGGGATATATTTCCTTTGCAAACTT. *GAPDH mRNA* primer is Fw- AAGAGGGATGCTGCCCTTAC and Rv- TACGGCCAAATCCGTTCACA.

### 2.10 Metabolomic analysis

Mice were euthanized via cervical dislocation. Inner ears including lateral walls were dissected and preserved at −80°C. Cochlear metabolites were extracted and analyzed by liquid chromatography–mass spectrometry (LC-MS) (LCMS-8060, Shimadzu Co., Tokyo, Japan). Peak detection was performed using multivariate data analysis software (Travers MS; Reifycs Inc., Tokyo, Japan). Data analyses included principal component analysis (PCA), heat mapping, enrichment analysis, pathway analysis, and network analysis using MetaboAnalyst^2^ (Pang et al., 2020).

### 2.11 Auditory brainstem responses (ABRs)

Auditory brainstem responses (ABRs) were recorded at 3, 6, and 12 months. Auditory stimuli were generated by an NI USB-6216 signal processor (National Instruments, Austin, TX, USA) consisting of 1-ms-tone bursts with a 0.2-ms-rise and fall time delivered at a rate of 14 per second. Stimuli were presented in a closed field using an MF-1 magnetic speaker (Tucker-Davis Technologies, Alachua, FL, USA). The polarities of the acoustic stimuli were altered to minimize the stimulus artifacts. Individual responses were amplified 20,000 times (one-slot ampbase; Melon Technos Co. Ltd. Kanagawa, Japan), digitally sampled at a rate of 10 kHz (NI USB-6216, National Instruments), and band-pass-filtered from 0.2 to 2 kHz. The responses are the averages of 500 individual responses to stimuli of the same frequency and intensity. The waveform was displayed using LabVIEW (National Instruments). The intensity-amplitude functions of the ABRs were obtained at each tested frequency (4, 8, 16, and 32 kHz) by varying the intensity of the tone bursts from 0 to 90 sound pressure levels (dB SPL) in 10 dB steps. ABR thresholds were defined as the minimum sound intensity necessary to elicit a distinguishable response (Miwa et al., 2023).

### 2.12 Distortion product otoacoustic emissions (DPOAEs)

The distortion product otoacoustic emissions (DPOAEs) were measured using an ER10B + probe microphone speaker system (Etymotic Research, Inc., Elk Grove Village, IL, USA) (Miwa et al., 2020, 2021). Stimuli with SPL1 = 75 dB and SPL2 = 65 dB were applied at a frequency ratio of f2/f1 = 1.22 across 4–20 kHz. Responses were analyzed using LabVIEW (National Instruments). Each recording was repeated ten times, and the recorded values were averaged.

### 2.13 Endocochlear potentials (EPs)

Endocochlear potentials (EPs) values were measured in scala media using heat-pulled microelectrodes filled with 150 mM KCl. Signals were amplified with an MEZ-7200 amplifier (Nihon Kohden, Tokyo, Japan) and recorded using LabVIEW (National Instruments) (Miwa et al., 2019, 2020).

### 2.14 Statistical analysis

Data were analyzed using GraphPad Prism Ver. 10.0.0. (GraphPad Software, San Diego, CA, USA). Statistical significance (*p* < 0.05) was determined using a one-way or two-way analysis of variance (ANOVA) with a *post hoc* Tukey test. Sample size and power analyses were conducted using Power and Sample Size Calculation software Ver. 3.1.6 (Biostatistics Department, Vanderbilt University, Nashville, TN, USA). Data are presented as mean ± standard error of the mean (SEM).

## 3 Results

### 3.1 MNAM supplementation in LFD-fed mice induces weight loss

Food and water consumption were similar in both LFD and LFD + MNAM groups. In both B6 and CBA strains, LFD-fed mice steadily gained weight throughout the 12-month observation period, whereas LFD + MNAM-fed mice exhibited significant weight loss (Supplementary Figure 2). Weight loss was evident at 6 (B6; *p* = 0.01) and 12 months (B6; *p* < 0.001, CBA; *p* < 0.001). Blood analyses revealed no significant differences between the two groups in both strains during the study period (Supplementary Figure 3).

### 3.2 MNAM diet accelerates ARHL in LFD-fed mice

**FIGURE 1:**
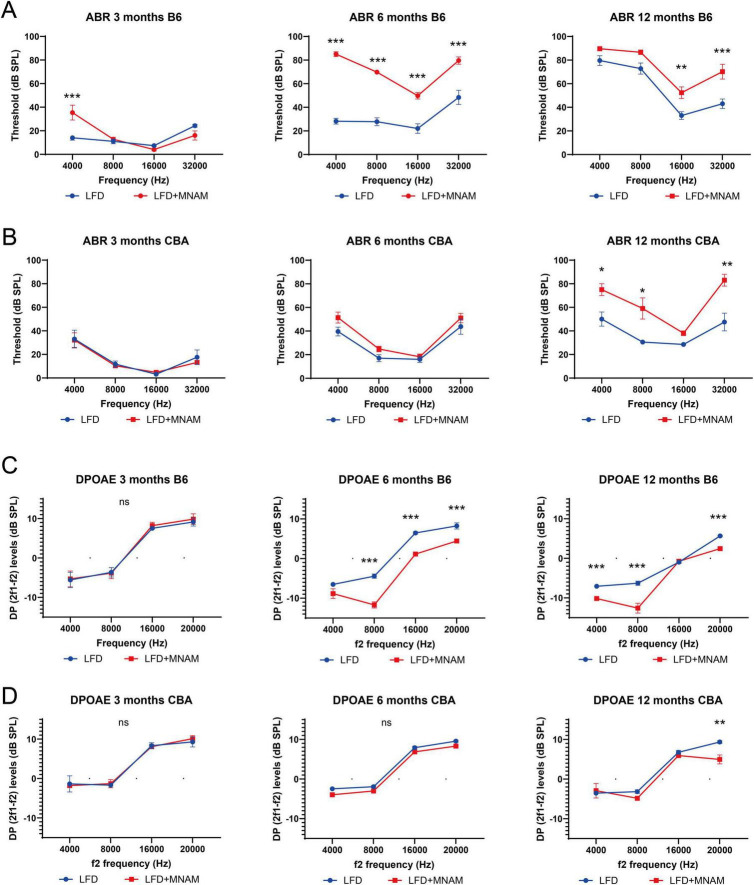
Onset of age-related hearing loss (ARHL) and the effect of MNAM supplementation on ARHL. **(A)** ABR recording results in the B6 strain: Auditory brainstem response (ABR) thresholds were recorded at 3, 6, and 12 months after commencement of MNAM supplementation. At 3 months, a threshold increase was observed only at 4 kHz in both LFD and LFD + MNAM groups. By 6 months, the mean thresholds for LFD + MNAM mice were significantly higher than those for LFD mice across all frequencies. At 12 months, the LFD + MNAM mice exhibited higher thresholds than the LFD mice at 16 and 32 kHz, indicating accelerated ARHL with MNAM supplementation in the B6 strain. **(B)** ABR recording results in the CBA strain: In the CBA strain, no differences in ABR thresholds were detected between LFD and LFD + MNAM mice at 3 months. Similarly, at 6 months, the threshold levels remained comparable between the groups. However, at 12 months, LFD + MNAM mice displayed significantly higher thresholds at 4, 8, and 32 kHz than did LFD mice, suggesting some susceptibility to MNAM-induced auditory threshold elevation at later stages. **(C)** DPOAE recording results in the B6 strain: Distortion product otoacoustic emissions (DPOAEs) were analyzed at 3, 6, and 12 months. At 3 months, no differences in DPOAE levels were detected between the LFD and LFD + MNAM groups across all frequencies. By 6 months, DPOAE levels in LFD + MNAM mice were significantly reduced compared to those in LFD mice at all frequencies except 4 kHz. At 12 months, reductions in DPOAE levels were observed at 4, 8, and 20 kHz in LFD + MNAM mice, indicating that cochlear dysfunction was induced by MNAM in the B6 strain. **(D)** DPOAE results in the CBA strain:In the CBA strain, no differences in DPOAE levels were detected between the LFD and LFD + MNAM groups at 3 months. At 6 months, although there was a slight reduction in DPOAE levels across all frequencies in the LFD + MNAM group, it did not reach statistical significance. By 12 months, the DPOAE levels at 20 kHz were significantly lower in the LFD + MNAM group, suggesting mild MNAM-induced cochlear impairment in the CBA strain. All groups, *n* = 5, *p < 0.05, **p < 0.01, ***p < 0.001, ns; not significant.

Auditory function was assessed using ABR thresholds. LFD + MNAM-fed B6 mice exhibited significantly higher ABR thresholds than LFD-fed B6 mice at 6 months (all frequencies, *p* < 0.001) and 12 months at 16 kHz (*p* = 0.003) and 32 kHz (*p* < 0.001) (Figure 1A). In CBA mice, threshold increases were observed at 12 months at 4 (*p* = 0.04), 8 (*p* = 0.02), and 32 kHz (*p* = 0.003) (Figure 1B).

DPOAE analysis revealed no differences in the outer hair cell function between the groups at 3 months for either strain (Figures 1C, D). By 6 months, DP levels in LFD + MNAM-fed B6 mice were significantly reduced at all frequencies except 4 kHz (*p* < 0.001), whereas CBA mice exhibited no differences in DP levels. At 12 months of age, DP levels were reduced at 4, 8, and 20 kHz in B6 mice (*p* < 0.001), and at 20 kHz in CBA mice (*p* = 0.006).

**FIGURE 2:**
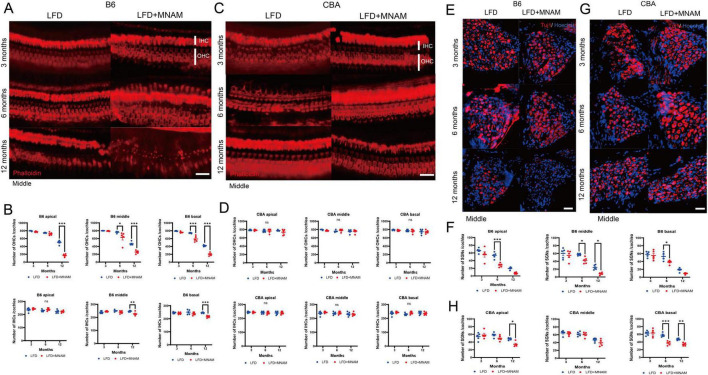
Effects of MNAM administration on hair cells (HCs) and spiral ganglion cells (SGCs). **(A)** Phalloidin staining of hair cells in the B6 strain: surface preparations from the middle turn of the cochlea revealed phalloidin-stained hair cells at 3, 6, and 12 months after initiating MNAM administration in B6 mice. **(B)** Hair cell counts in B6 mice: quantification of outer hair cells (OHCs) and inner hair cells (IHCs) in all cochlear turns showed no differences between the LFD and LFD + MNAM groups at 3 months. However, after 6 months, a significant decrease in OHCs was observed in the middle and basal turns of LFD + MNAM mice. For IHCs, no differences were detected at 3 or 6 months; however, by 12 months, the LFD + MNAM mice exhibited a reduction in the middle and basal turns. **(C)** Phalloidin staining of hair cells in the CBA strain: hair cells in the middle turn of the cochlea of CBA mice were visualized using phalloidin staining at 3, 6, and 12 months after the start of the experiment. **(D)** Hair Cell Counts in CBA Mice: In the CBA strain, there were no differences in the counts of OHCs or IHCs between the LFD and LFD + MNAM groups across all cochlear turns at any time point examined, indicating that MNAM administration did not affect hair cell survival. **(E)** Immunostaining of spiral ganglion cells in the B6 strain anti-Tuj1 antibody staining was used to visualize SGCs in the middle turn 3, 6, and 12 months after MNAM administration in B6 mice. **(F)** SGC counts in B6 mice: At 3 months of age, no differences were detected in the number of SGCs between the LFD and LFD + MNAM groups. By 6 months, a significant reduction in SGC counts was observed in all cochlear turns in the LFD + MNAM group compared to the LFD group. At 12 months, the LFD + MNAM group exhibited a significant reduction in SGC number in the middle turn, with decreases observed across all turns. **(G)** Immunostaining of Spiral Ganglion Cells in the CBA Strain: SGCs in the middle turn of CBA mice were visualized with anti-Tuj1 antibody staining at 3, 6, and 12 months following MNAM administration. **(H)** SGC Counts in CBA Mice: In the CBA strain, no differences in SGC counts were detected at 3 months of age. At 6 months, a significant reduction was observed only in the basal turn of the cochlea in the LFD + MNAM group. At 12 months, the SGC counts were reduced in all cochlear turns, with significant differences specifically noted in the apical and basal turns. All groups, *n* = 5, *p < 0.05, **p < 0.01, ***p < 0.001, ns; not significant. Bars: 100 μm; MNAM; N^1^-methylnicotinamide; OHCs, outer hair cells; IHCs, inner hair cells; SGCs, spiral ganglion cells; anti-Tuj1, anti-beta III tubulin.

Hair cell counts at 3 months were similar in LFD and LFD + MNAM-fed B6 mice; however, at 12 months, OHC counts were significantly reduced in the middle and basal cochlear turns (*p* = 0.02 and *p* < 0.001, respectively). The IHC counts followed a similar pattern (Figures 2A, B). Moreover, we observed no significant differences in hair cell counts across all cochlear regions in CBA mice (Figures 2C, D).

SGC counts at 3 months were similar in LFD and LFD + MNAM B6 mice. At 6 months, SGC counts were lower in LFD + MNAM-fed mice across all cochlear regions (apical, *p* < 0.001; middle, *p* = 0.03; basal, *p* = 0.01). At 12 months, we observed significantly reduced SGC counts only in the middle cochlear turn (*p* = 0.02) (Figures 2E, F). In CBA mice, SGC reduction was evident at 6 months in the basal turn (*p* < 0.001) and at 12 months in both the apical (*p* = 0.02) and basal turns (*p* = 0.008) (Figures 2G, H).

### 3.3 EPs decrease in MNAM-fed mice

**FIGURE 3:**
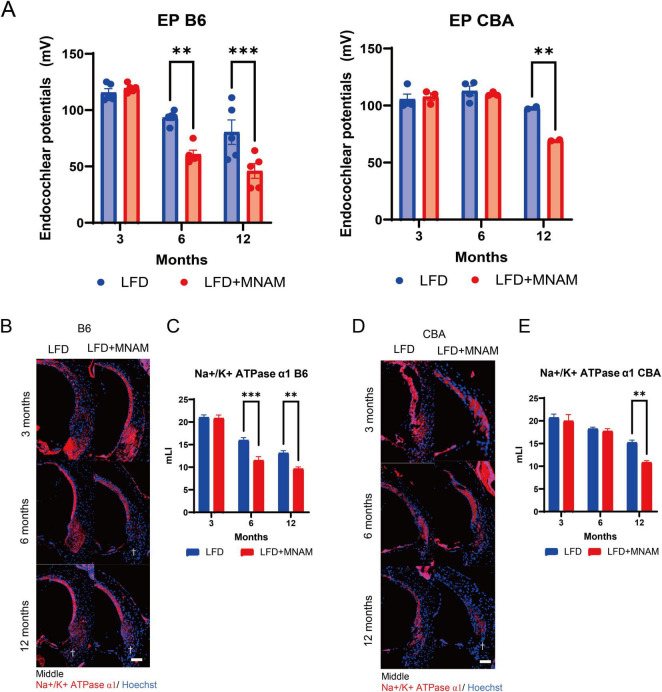
Effects of MNAM administration on endocochlear potentials and tissue staining. **(A)** EPs in B6 and CBA Strains: Average endocochlear potentials (Eps) were measured at 3, 6, and 12 months after the initiation of MNAM administration. At 3 months, no significant differences in either the B6 or CBA strains were observed between the LFD and LFD + MNAM groups. By 6 months, the average EPs in LFD + MNAM mice were significantly lower than those in LFD mice of the B6 strain, whereas no differences were noted in the CBA strain. At 12 months, the overall EPs decreased across all groups, with the MNAM-administered mice in both the B6 and CBA strains showing a more pronounced reduction than their respective controls. **(B)** Immunostaining of Na^+^/K^+^-ATPase α1 in the Spiral Ligament (SLi): Immunostaining for Na^+^/K^+^-ATPase α1 was performed in Type II and IV fibrocyte regions of the SLi at 6 months post-experiment initiation. Reduced expression of Na^+^/K^+^-ATPase α1 was evident in LFD + MNAM mice compared to LFD mice, as indicated by a dagger symbol in the figure. **(C)** Quantitative Analysis of Na^+^/K^+^-ATPase α1 Expression in B6 Mice: At 3 months, Na^+^/K^+^-ATPase α1 was expressed in Type II and IV fibrocytes of the spiral ligament without significant differences in staining intensity between LFD and LFD + MNAM groups. At 6 months, Na^+^/K^+^-ATPase α1 expression in the B6 strain was markedly reduced in the MNAM-treated group. At 12 months, a significant reduction in expression was observed in the MNAM-treated group compared to that in the control group. **(D)** Immunostaining of Na^+^/K^+^-ATPase α1 in the Spiral Ligament of CBA Mice: Similar immunostaining was performed for Na^+^/K^+^-ATPase α1 in the CBA strain. At 3 months, Na^+^/K^+^-ATPase α1 expression in Type II and IV fibrocytes showed no differences in staining intensity between the two groups. At 6 months, only a slight reduction in staining intensity was observed in MNAM-treated mice. By 12 months, the MNAM-treated group exhibited a significant reduction in Na^+^/K^+^-ATPase α1 expression, as indicated by a dagger symbol in the figure. **(E)** Quantitative Analysis of Na^+^/K^+^-ATPase α1 Expression in CBA Mice: Quantitative evaluation of staining intensity revealed no significant differences at 3 months. At 6 months, a minor decrease in Na^+^/K^+^-ATPase α1 expression was observed in MNAM-treated mice. However, at 12 months, a significant reduction in expression intensity was evident in the MNAM-administered group. All groups, *n* = 5, **p < 0.01, ***p < 0.001. Bars: 100 μm; MNAM: N^1^-methylnicotinamide.

We observed no significant differences in EPs at 3 months in either strain (Figure 3A). By 6 months, the EP values in LFD + MNAM-fed B6 mice were significantly reduced when compared with those of LFD-fed mice (*p* = 0.001), with similar trends at 12 months in both the B6 (*p* < 0.001) and CBA strains (*p* = 0.003).

Immunohistochemical staining for Na^+^/K^+^ ATPase α1 in the SLi at 3 months revealed no differences (Figures 3B–E). Na^+^/K^+^ ATPase α1 expression at 6 months was significantly reduced in LFD + MNAM-fed B6 mice (*p* < 0.001), and only minor reductions were observed in CBA mice. By 12 months, significant reductions in Na^+^/K^+^ ATPase α1 were observed in both strains (*p* = 0.001).

### 3.4 MNAM increases SIRT1 expression

**FIGURE 4:**
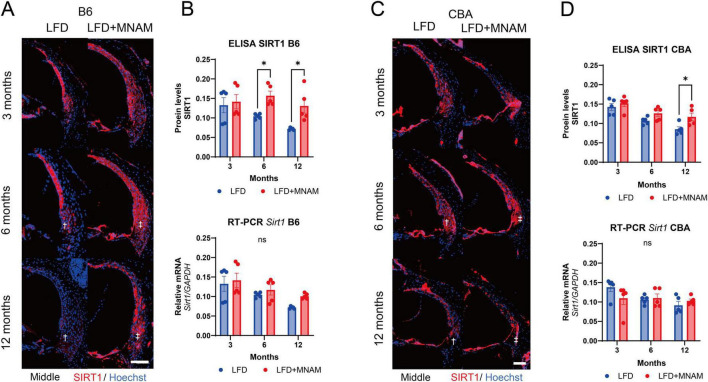
Expression of SIRT1 protein and *Sirt1* mRNA in the cochlea. **(A)** SIRT1 Staining in the Cochlea of B6 Mice: Immunofluorescent staining of SIRT1 (red) was performed in the middle turn of the cochlea at 3, 6, and 12 months after the start of MNAM administration. SIRT1 is predominantly localized in the outer hair cells (OHCs) and Type II and IV fibrocytes of the spiral ligament (SLi) across all cochlear turns in B6 mice. In LFD mice, SIRT1 expression decreased with age, particularly in the SLi, as indicated by the daggered symbol. However, in MNAM-treated mice, SIRT1 expression did not decline, suggesting the preservation of protein levels with MNAM supplementation, as indicated by the double daggered symbol. **(B)** Quantitative Analysis of SIRT1 Protein and *Sirt1* mRNA in B6 Mice: SIRT1 protein expression in the cochlea was performed using ELISA at 3, 6, and 12 months of age. In LFD mice, SIRT1 protein levels progressively decreased with age. In contrast, MNAM-treated mice showed significantly higher SIRT1 expression at 6 and 12 months than LFD mice. *Sirt1* mRNA levels were assessed using qRT-PCR, with GAPDH as the reference gene. No significant differences in Sirt1 mRNA expression were observed between the LFD and LFD + MNAM groups at any time point, indicating that MNAM’s effect of MNAM on SIRT1 protein levels may be post-transcriptional. **(C)** SIRT1 Staining in the Cochlea of CBA Mice: Similar immunofluorescent staining was conducted in CBA mice at 3, 6, and 12 months. SIRT1 was prominently localized in OHCs and Type II and IV fibrocytes of the SLi across all cochlear turns. In LFD mice, SIRT1 expression decreases with age, particularly in the SLi. However, MNAM-treated mice maintained SIRT1 expression over time, mirroring the results observed in B6 mice. **(D)** Quantitative Analysis of SIRT1 Protein and mRNA in CBA Mice: In CBA mice, SIRT1 protein expression, measured by ELISA, declined with age in the LFD group. However, MNAM treatment preserved SIRT1 protein levels, with significantly higher expression observed at 6 and 12 months compared to that in LFD mice. *Sirt1* mRNA levels, evaluated using qRT-PCR, showed no significant differences between LFD and LFD + MNAM groups at any stage, suggesting a similar post-transcriptional regulation mechanism in the CBA strain. All groups, *n* = 5, *p < 0.05, ns; not significant. Bars: 100 μm.

Immunohistochemical analysis revealed that SIRT1 is present in cochlear SLi cells (types I, II, and V). SIRT1 expression decreased with age in LFD-fed mice; however, MNAM supplementation increased SIRT1 levels (Figure 4A). ELISA confirmed significantly higher cochlear SIRT1 protein levels in LFD + MNAM-fed mice at 6 (*p* = 0.04) and 12 months (*p* = 0.02) (Figure 4B). Notably, RT-PCR revealed no differences in SIRT1 mRNA expression between groups at any stage, which indicates post-transcriptional regulation (Figure 4B). CBA mice exhibited similar trends in SIRT1 expression (Figures 4C, D; *p* = 0.04 at 12 months).

### 3.5 Metabolomic analysis revealed differences in metabolic pathways in MNAM-treated mice

**FIGURE 5:**
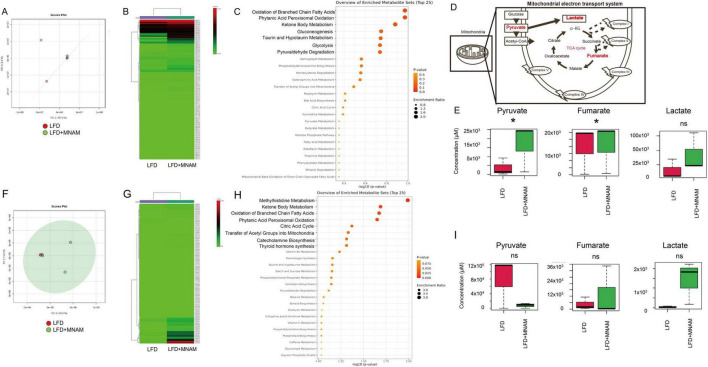
Metabolome heatmap, enrichment analysis, and principal component analysis (PCA). **(A)** PCA Analysis (B6 Mice): Principal component analysis (PCA) comparing the cochleae of LFD and LFD + MNAM mice at 6 months revealed distinct metabolic profiles between the two groups. **(B)** Heatmap Analysis (B6 Mice): Heatmap analysis at six months showed significant differences in the metabolomic profiles between the LFD and LFD + MNAM groups, highlighting changes in pathways associated with mitochondrial function and energy metabolism. **(C)** Enrichment Analysis (B6 Mice): Enrichment analysis identified the selective activation of pathways related to the oxidation of short-chain saturated fatty acids, phthalic acid peroxidation, ketone body metabolism, gluconeogenesis, and mitochondrial metabolism in LFD + MNAM mice compared with LFD controls. **(D)** Schema of the TCA Cycle: A schematic representation of the tricarboxylic acid (TCA) cycle is included to illustrate the metabolic pathways affected by MNAM administration. **(E)** Analysis (B6 Mice) Key TCA cycle intermediates, including pyruvate, fumarate, and lactate, were analyzed. In LFD + MNAM mice, all three metabolites showed increased levels compared to those in LFD controls, suggesting enhanced mitochondrial oxidation and metabolic activity in the cochlea. **(F)** PCA Analysis (CBA Mice): PCA comparing the cochleae of LFD and LFD + MNAM mice at six months revealed distinct metabolic profiles in the CBA strain, further supporting the strain-independent metabolic effects of MNAM. **(G)** Heatmap Analysis (CBA Mice): A heatmap analysis at 6 months identified differences in the metabolomic profiles between the LFD and LFD + MNAM groups in the CBA strain, consistent with observations in the B6 strain. **(H)** Enrichment Analysis (CBA Mice) Enrichment analysis in CBA mice confirmed the activation of metabolic pathways related to methylhistidine metabolism, short-chain saturated fatty acid oxidation, phthalic acid peroxidation, ketone body metabolism, gluconeogenesis, and mitochondrial function in the MNAM-treated group. **(I)** Pathway Analysis (CBA Mice) Analysis of TCA cycle metabolites revealed changes in fumarate, lactate, and pyruvate levels. In the LFD + MNAM mice, pyruvate levels decreased, whereas fumarate and lactate accumulated. Although these trends were noted, the statistical analysis did not show significant differences between the groups. All groups, *n* = 5, *p < 0.05, ns; not significant.

To investigate MNAM-induced metabolic changes, metabolome concentration analysis was conducted at six months, the stage when auditory differences were most significant. In the B6 strain, PCA and heatmap analyses revealed distinct metabolic profiles in the LFD and LFD + MNAM groups (Figures 5A, B). Enrichment analysis identified selectively activated pathways including short-chain fatty acid oxidation, phthalic acid peroxidation, ketone body metabolism, gluconeogenesis, and mitochondrial metabolism (Figure 5C).

Analysis of the TCA cycle revealed increased levels of pyruvate (*p* = 0.02), fumaric acid (*p* = 0.04), and lactic acid in LFD + MNAM mice when compared with those of LFD mice. This accumulation suggested TCA cycle dysfunction and metabolic substrate build-up (Figures 5D, E).

In the CBA strain, similar differences were observed in PCA and heatmap analyses (Figures 5F, G). The pathways associated with methylhistidine metabolism and mitochondrial processes were selectively activated (Figure 5H). In the TCA cycle, pyruvate levels decreased, whereas fumaric acid and lactic acid accumulated; however, these changes were not statistically significant (Figures 5D, I).

The key metabolites identified through heatmap and network analyses in B6 mice at 6 months included serine, orotate, threonine, and guanylic acid (Supplementary Figures 4A, B). In the CBA strain, the key metabolites were UDP-glucose, hydroxybenzoic acid, homocysteine, cysteine, threonine, adenosine, and lactate (Supplementary Figures 4C, D).

Metabolomic analysis at 12 months in B6 mice yielded similar results, with no significant differences in the TCA cycle between the LFD and LFD + MNAM groups (Supplementary Figures 5A–D). The key metabolites at 12 months of age included guanosine, citrulline, hydroxyproline, and homoprotocatechuate (Supplementary Figures 5B, E).

This analysis highlights the role of MNAM in the modulation of mitochondrial and energy metabolism, which may contribute to the observed auditory and physiological changes.

## 4 Discussion

### 4.1 MNAM supplementation increases SIRT1 expression in the cochlea and accelerates AHRL progression

Our findings demonstrate that MNAM supplementation in LFD-fed mice accelerates ARHL by damaging the cochlear sensory organ, lateral wall, and overall cochlear function. These findings contrast with earlier research that indicated that in HFD-fed B6 mice, MNAM protects the cochlea and preserves cochlear function, thereby delaying ARHL onset (Miwa, 2021).

Interestingly, both B6 and CBA mice fed LFD + MNAM exhibited early-onset ARHL, with B6 mice demonstrating a more rapid progression. In B6 mice, the OHCs in the cochlear sensory organs were significantly damaged, whereas in CBA mice, the outer hair cells remained relatively intact. Furthermore, in CBA mice, at the 6-month mark, the EPs remained unaffected, and lateral wall damage was mild. This suggests slower aging in CBA mice than in B6 mice, which is consistent with previous findings (Keithley et al., 2004; Fujita et al., 2015; Kim et al., 2019). However, the observed cochlear damage in CBA mice indicates that MNAM has an impact, albeit less pronounced than that in B6 mice.

As observed in earlier studies, MNAM increased SIRT1 protein expression without affecting *Sirt1* mRNA levels in the cochlea, likely because of stabilization of the SIRT1 protein (Hong et al., 2015; Miwa, 2021). Immunohistochemical staining revealed that SIRT1 expression, which decreased with age in LFD-fed mice, was preserved or increased in LFD + MNAM-fed mice. This trend was observed in both B6 and CBA mice, although the SIRT1 decline with age was more gradual in CBA mice, which is consistent with their slower aging characteristics.

Metabolomic analysis at six months of age revealed the activation of specific metabolic pathways in the cochlea of LFD + MNAM-fed B6 mice. These include pathways related to the oxidation of short-chain fatty acids, phthalic acid peroxidation, ketone body metabolism, gluconeogenesis, and mitochondrial metabolism. SIRT1 regulates central metabolic functions such as lipogenesis, protein synthesis, gluconeogenesis, and metabolic homeostasis through deacetylation (García-Rodríguez et al., 2014). Analysis of TCA cycle intermediates suggested dysfunctional energy metabolism in the LFD + MNAM mice, with increased levels of pyruvate, fumaric acid, and lactic acid. Key metabolites, such as serine, orotate, threonine, and guanylic acid, which are involved in the protection and repair of mitochondria, cell division, and immunity (Miura et al., 1979; Nelson et al., 1992), were also elevated, which reflects potential compensatory mechanisms for tissue damage that ultimately fail and lead to cochlear dysfunction. Mice at 12 months demonstrated similar findings, with metabolites, such as guanosine, citrulline, hydroxyproline, and homoprotocatechuate, indicating oxidative stress and tissue deterioration (Ito et al., 1980; Blank et al., 2010; Dong et al., 2013; Bettio et al., 2016). At 12 months, there was no significant difference between the MNAM-treated and non-treated groups due to accelerated aging.

In CBA mice, metabolomic analysis revealed the activation of oxidative pathways and selective metabolic changes. However, TCA cycle analysis revealed no significant differences between the groups, which suggests that the metabolic pathways in CBA mice remained functional for longer periods than those in B6 mice.

### 4.2 Optimal SIRT1 expression may be critical for preventing ARHL

Our results suggest that, while moderate SIRT1 expression may protect against ARHL, overexpression induced by MNAM exacerbates this condition. HFD-fed mice exhibited reduced SIRT1 expression, which was mitigated by MNAM supplementation, resulting in delaying ARHL onset (Miwa, 2021). Additionally, *Sirt1*-deficient mice develop hearing loss (Cheng et al., 2003), and heterozygous *Sirt1* transgenic B6 mice exhibit reduced oxidative damage and slower ARHL progression (Han et al., 2016). These findings, combined with those of the present study, suggest that in cochlear cells, SIRT1, when overexpressed, acts as an aging accelerator.

This implies a dual role for SIRT1; minimal expression levels protect against ARHL, whereas overexpression promotes it. Further studies, including genetic models of SIRT1 overexpression, are required to confirm this hypothesis. Alternatively, optimizing the concentration of MNAM and performing further experiments may provide deeper insights.

### 4.3 Limitations

This study had several limitations. First, while MNAM-induced SIRT1 overexpression was demonstrated to increase SIRT1 protein levels, its direct effects on hearing loss remain unclear. In this study, the potential cochlear damage associated with MNAM exposure and the cochlear damage potentially resulting from SIRT1 overexpression are intertwined. MNAM may negatively influence other pathways, such as Nnmt-related metabolism (Liu et al., 2015; Wang et al., 2022). Further investigation is warranted to distinguish and verify these independent mechanisms in future research. Second, we did not investigate the role of SIRT3 (Brown et al., 2014), which is also regulated by MNAM. Third, due to budget constraints, we performed the experiments sequentially rather than simultaneously, which limited our ability to study all groups —such as HFD, LFD, genetically modified mice, and different strains—under uniform conditions. Third, we did not investigate the molecular mechanisms underlying the association between SIRT1 overexpression and cochlear damage. Although SIRT1 may act through pathways involving Foxo3a in hair cells (Han et al., 2016), its role in the lateral wall of the cochlea remains unclear. Future research should focus on elucidating the downstream cascades of SIRT1 in cochlear aging and damage to provide deeper mechanistic insights. Finally, in our current study, we observed that the MNAM-treated group exhibited elevated auditory thresholds in the low-frequency range as measured by ABR. In contrast, the DPOAE and histological findings did not show consistent changes, making interpretation challenging. However, given previous reports suggesting that certain drugs tend to accumulate in the cochlear apex following administration (Sadreev et al., 2019), it is hypothesized that MNAM may similarly accumulate in the apex, potentially impairing cochlear functions such as EP.

## 5 Conclusion

In LFD-fed mice, MNAM supplementation accelerates ARHL by damaging the cochlear sensory organs, lateral wall, and cochlear function. These findings suggest that excessive MNAM administration induces overexpression of SIRT1 protein, which disrupts the metabolic pathways critical for auditory function in the aging cochlea.

In conjunction with earlier findings, our results indicate that a *nominal* expression level of SIRT1 may play a protective role in preventing ARHL progression, whereas *overexpression* exacerbates ARHL. Future studies should focus on determining the optimal SIRT1 expression level required to prevent ARHL and identifying strategies to maintain this balance in aging cochlear cells.

## Data Availability

The raw data supporting the conclusions of this article will be made available by the authors, without undue reservation.

## Footnotes

1
https://www.mousephenotype.org

2
https://www.metaboanalyst.ca/

## Supplementary material

Supplementary Figure 1 Cascade of Sirtuin 1 (Sirt1) activation. SIRT 1, Sirtuin 1; NMN, nicotinamide mononucleotide; NAD, nicotinamide adenine dinucleotide; Nnmt, nicotinamide N-methyltransferase; MNAM, N^1^-methylnicotinamide.

Supplementary Figure 2 Effect of MNAM on body weight. **(A,B)** Body weight changes were monitored in B6 **(A)** and CBA **(B)** mice over a 12-month observation period. In both strains, mice fed a low-fat diet (LFD) exhibited a steady increase in body weight throughout the study. In contrast, mice fed an LFD supplemented with MNAM (LFD + MNAM) showed a notable decrease in body weight compared to the LFD group in both strains, suggesting that MNAM supplementation may influence metabolic processes that regulate weight. All groups, *n* = 5, **p* < 0.05, ****p* < 0.001.

Supplementary Figure 3 Effect of MNAM on blood test results. **(A,B)** Blood analysis was conducted to evaluate the systemic effects of MNAM administration in B6 **(A)** and CBA **(B)** mice. No significant differences were observed between the LFD and LFD + MNAM groups in either strain, indicating that MNAM supplementation did not cause notable changes in blood parameters over the course of the study. All groups, *n* = 5, ns; not significant.

Supplementary Figure 4 Key Metabolites Identified in Metabolome Analysis of MNAM Effects. **(A)** The top 25 metabolites identified in the heatmap analysis revealed serine and orotate to be key metabolites associated with the effect of MNAM in the B6 strain at the 6-month stage. **(B)** Network analysis further identified threonine and guanylic acid as significant metabolites in the B6 strain at the 6-month stage, suggesting their involvement in pathways influenced by MNAM administration. **(C)** In the CBA strain, heatmap analysis highlighted UDP-glucose as a key metabolite detected at the 6-month stage, indicating a distinct metabolic response to MNAM.

Supplementary Figure 5 Metabolome analysis at 12 months in B6 strain mice. **(A)** PCA Analysis: Principal component analysis (PCA) revealed distinct clustering of metabolic profiles, indicating metabolic differences between the LFD and LFD + MNAM groups at the 12-month stage. **(B)** Heatmap Analysis: The top 25 metabolites identified in the heatmap analysis highlighted guanosine and citrulline as key metabolites associated with MNAM administration, suggesting their involvement in the metabolic pathways affected by MNAM. **(C)** Enrichment Analysis: Enrichment analysis revealed significant alterations in metabolic pathways influenced by MNAM, further emphasizing its impact on cochlear metabolism. **(D)** TCA Cycle Analysis: No significant differences were observed in TCA cycle metabolites between the LFD and LFD + MNAM groups, suggesting that MNAM did not markedly alter this fundamental metabolic pathway in the B6 strain at 12 months. **(E)** Network Analysis: Network analysis identified hydroxyproline and homoprotocatechuate as key metabolites, highlighting their potential roles in the pathways modulated by MNAM in aged B6 mice. All groups, *n* = 5, ns; not significant.
