# Supplementary figures and images for: N1-methylnicotinamide promotes age-related cochlear damage via the overexpression of SIRT1
Source: Front Cell Neurosci. 2025 Jan 31;19:1542164. doi: 10.3389/fncel.2025.1542164 (PMC11825784; doi:10.3389/fncel.2025.1542164)

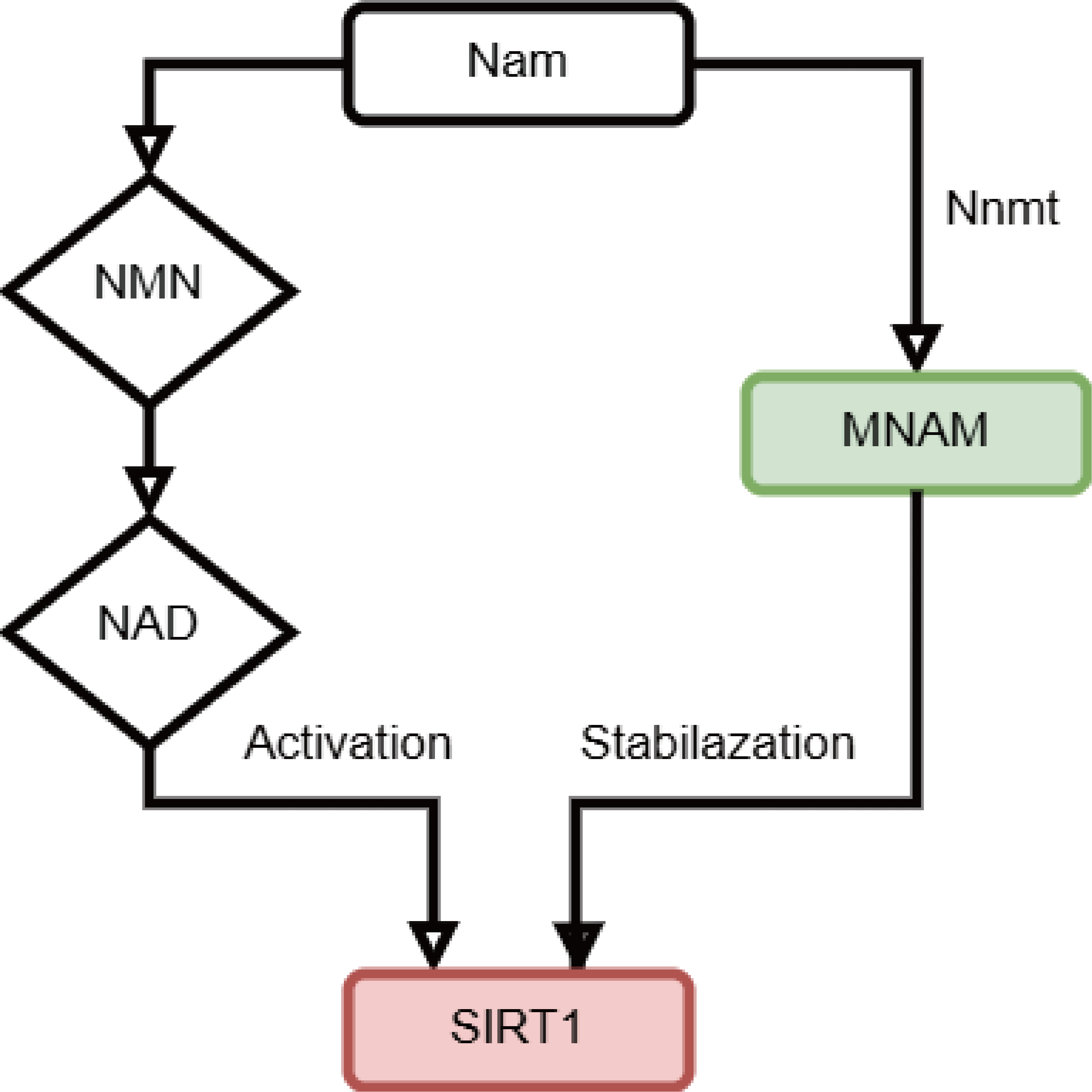

Supplement: Supplementary Figure 1 — Cascade of Sirtuin 1 (Sirt1) activation. SIRT 1, Sirtuin 1; NMN, nicotinamide mononucleotide; NAD, nicotinamide adenine dinucleotide; Nnmt, nicotinamide N-methyltransferase; MNAM, N1-methylnicotinamide. [file Image_1.tif]

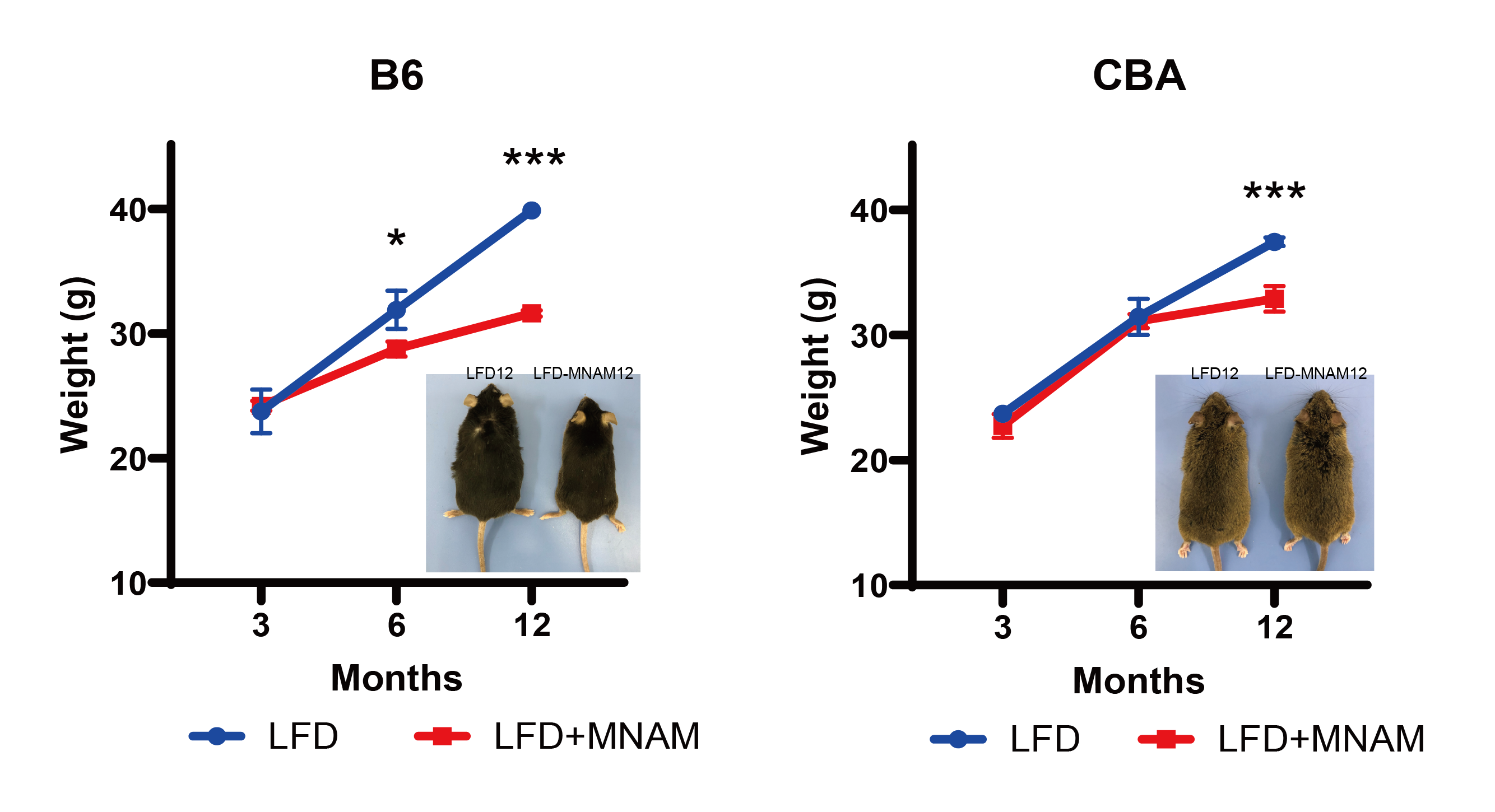

Supplement: Supplementary Figure 2 — Effect of MNAM on body weight. (A,B) Body weight changes were monitored in B6 (A) and CBA (B) mice over a 12-month observation period. In both strains, mice fed a low-fat diet (LFD) exhibited a steady increase in body weight throughout the study. In contrast, mice fed an LFD supplemented with MNAM (LFD + MNAM) showed a notable decrease in body weight compared to the LFD group in both strains, suggesting that MNAM supplementation may influence metabolic processes that regulate weight. All groups, n = 5, *p < 0.05, ***p < 0.001. [file Image_2.tif]

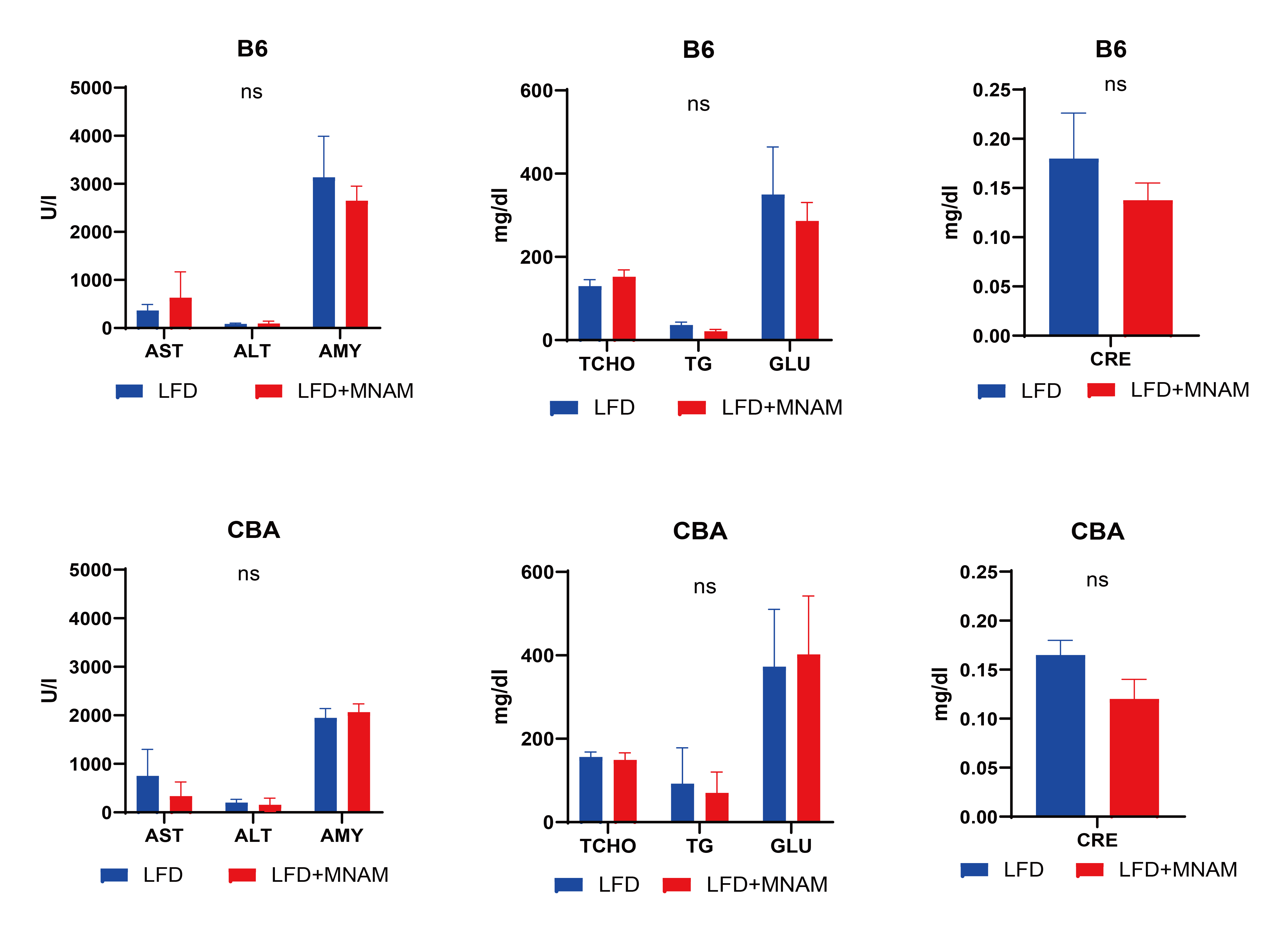

Supplement: Supplementary Figure 3 — Effect of MNAM on blood test results. (A,B) Blood analysis was conducted to evaluate the systemic effects of MNAM administration in B6 (A) and CBA (B) mice. No significant differences were observed between the LFD and LFD + MNAM groups in either strain, indicating that MNAM supplementation did not cause notable changes in blood parameters over the course of the study. All groups, n = 5, ns; not significant. [file Image_3.tif]

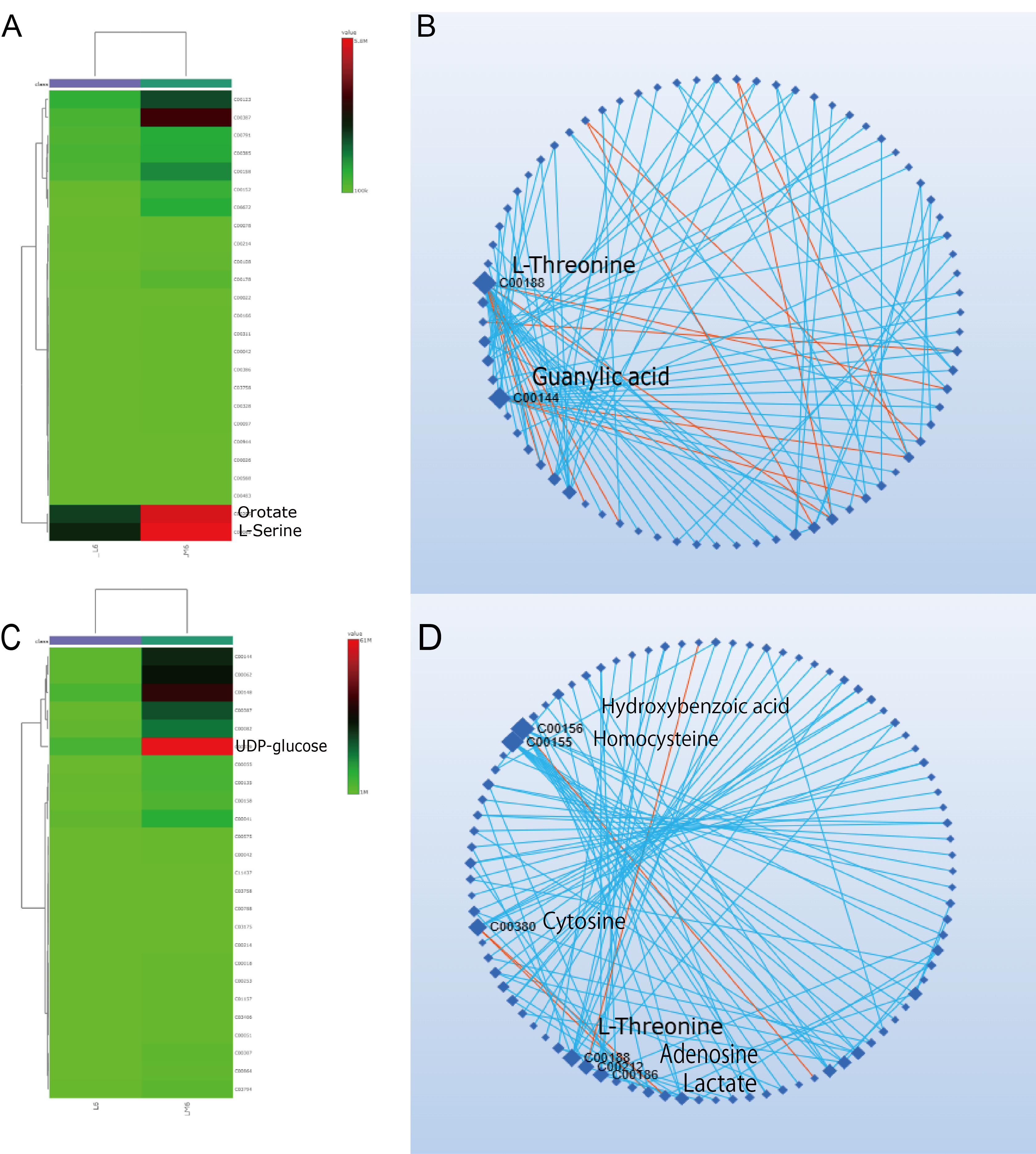

Supplement: Supplementary Figure 4 — Key Metabolites Identified in Metabolome Analysis of MNAM Effects. (A) The top 25 metabolites identified in the heatmap analysis revealed serine and orotate to be key metabolites associated with the effect of MNAM in the B6 strain at the 6-month stage. (B) Network analysis further identified threonine and guanylic acid as significant metabolites in the B6 strain at the 6-month stage, suggesting their involvement in pathways influenced by MNAM administration. (C) In the CBA strain, heatmap analysis highlighted UDP-glucose as a key metabolite detected at the 6-month stage, indicating a distinct metabolic response to MNAM. [file Image_4.tif]

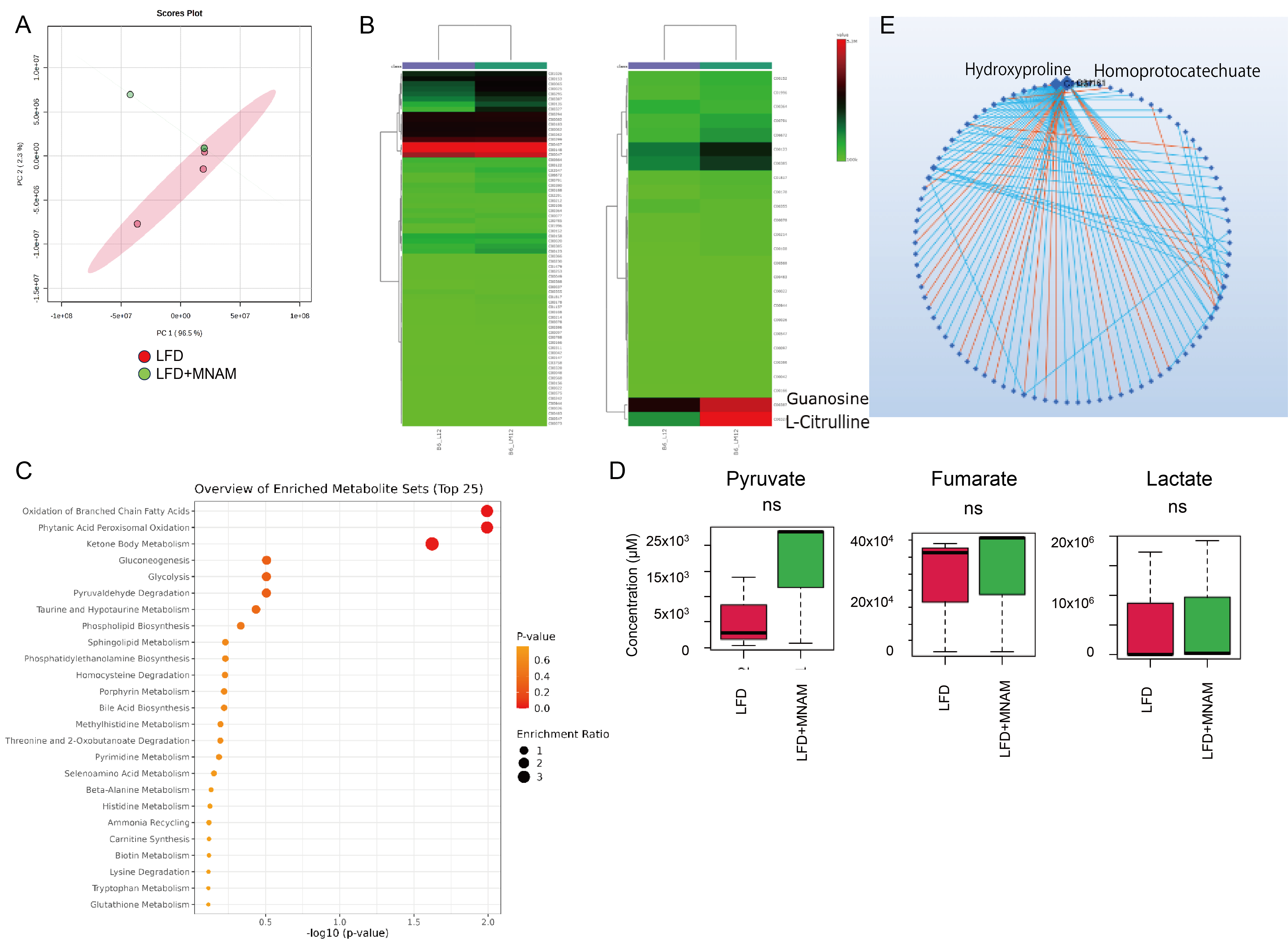

Supplement: Supplementary Figure 5 — Metabolome analysis at 12 months in B6 strain mice. (A) PCA Analysis: Principal component analysis (PCA) revealed distinct clustering of metabolic profiles, indicating metabolic differences between the LFD and LFD + MNAM groups at the 12-month stage. (B) Heatmap Analysis: The top 25 metabolites identified in the heatmap analysis highlighted guanosine and citrulline as key metabolites associated with MNAM administration, suggesting their involvement in the metabolic pathways affected by MNAM. (C) Enrichment Analysis: Enrichment analysis revealed significant alterations in metabolic pathways influenced by MNAM, further emphasizing its impact on cochlear metabolism. (D) TCA Cycle Analysis: No significant differences were observed in TCA cycle metabolites between the LFD and LFD + MNAM groups, suggesting that MNAM did not markedly alter this fundamental metabolic pathway in the B6 strain at 12 months. (E) Network Analysis: Network analysis identified hydroxyproline and homoprotocatechuate as key metabolites, highlighting their potential roles in the pathways modulated by MNAM in aged B6 mice. All groups, n = 5, ns; not significant. [file Image_5.tif]
